# Suppressing spontaneous polarization of p-GaN by graphene oxide passivation: Augmented light output of GaN UV-LED

**DOI:** 10.1038/srep07778

**Published:** 2015-01-14

**Authors:** Hyun Jeong, Seung Yol Jeong, Doo Jae Park, Hyeon Jun Jeong, Sooyeon Jeong, Joong Tark Han, Hee Jin Jeong, Sunhye Yang, Ho Young Kim, Kang-Jun Baeg, Sae June Park, Yeong Hwan Ahn, Eun-Kyung Suh, Geon-Woong Lee, Young Hee Lee, Mun Seok Jeong

**Affiliations:** 1 Center for Integrated Nanostructure Physics (CINAP), Institute for Basic Science (IBS), Sungkyunkwan University, Suwon 440-746, Republic of Korea; 2 Graphene Hybrid World Class Laboratory, Nano Carbon Materials Research Group, Korea Electrotechnology Research Institute, Changwon 641-120, Republic of Korea; 3 Department of Physics and Division of Energy Systems Research, Ajou University, Suwon 443-749, Republic of Korea; 4 School of Semiconductor and Chemical Engineering, Semiconductor Physics Research Center, Chonbuk National University, Jeonju 561-756, Republic of Korea; 5 Laboratoire de Nanotechnologie et d'Instrumentation Optique, Institut Charles Delaunay, CNRS-UMR 6279, Université de Technologie de Troyes, BP 2060, 10010 Troyes, France; 6 Department of Energy Science, Sungkyunkwan University, Suwon 440-746, Republic of Korea

## Abstract

GaN-based ultraviolet (UV) LEDs are widely used in numerous applications, including white light pump sources and high-density optical data storage. However, one notorious issue is low hole injection rate in p-type transport layer due to poorly activated holes and spontaneous polarization, giving rise to insufficient light emission efficiency. Therefore, improving hole injection rate is a key step towards high performance UV-LEDs. Here, we report a new method of suppressing spontaneous polarization in p-type region to augment light output of UV-LEDs. This was achieved by simply passivating graphene oxide (GO) on top of the fully fabricated LED. The dipole layer formed by the passivated GO enhanced hole injection rate by suppressing spontaneous polarization in p-type region. The homogeneity of electroluminescence intensity in active layers was improved due to band filling effect. As a consequence, the light output was enhanced by 60% in linear current region. Our simple approach of suppressing spontaneous polarization of p-GaN using GO passivation disrupts the current state of the art technology and will be useful for high-efficiency UV-LED technology.

GaN-based LED consists of active quantum wells, electron and hole transport layers, and contact metals^1^,^2^. One technological bottleneck in GaN LED is strong spontaneous electric polarization formed in p-GaN (hole transport layer), leading to poor hole injection rate, although heavily p-doped GaN has been successfully grown on c-plane sapphire substrates^3^,^4^. Owing to this polarization, hole carriers in p-GaN are locally bound by potential gradient, preventing them from contributing to the radiative recombination in multiple quantum wells (MQWs)^5^. To further improve light output power, increase of hole injection rate by suppression of spontaneous electric polarization is mandatory^6^. Graphene oxide (GO) nanosheets possess numerous oxygen functional groups and therefore may form strong dipole layers to modify such electric polarization^7^,^8^,^9^,^10^. In this study, we propose a simple and effective method to suppress spontaneous polarization of p-GaN transport layer by simply spin-casting GO layers on the conventionally fabricated UV-LED to eventually enhance the light output power.

## Results and discussion

**Figure 1:**
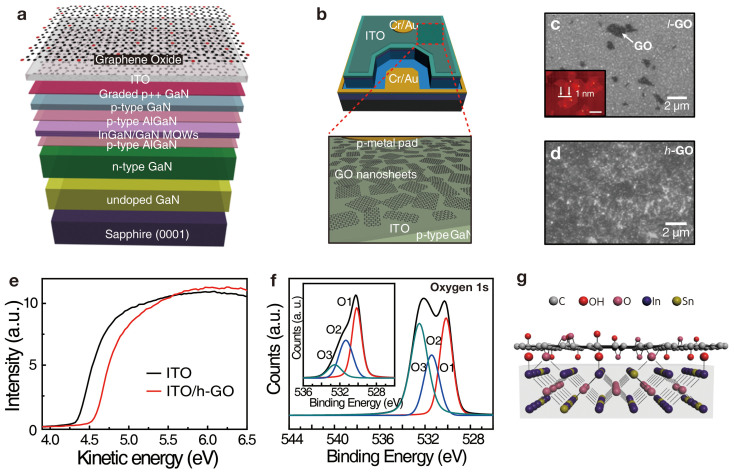
Preparation and characterization of GO-passivated UV-LEDs. (a) Schematic of the GaN-based UV-LED structure grown by MOCVD, and (b) fully fabricated device covered with GO nanosheets. Field-emission scanning electron microscopy images of the ITO surface (c) without and (d) with hydrophilic treatment. The inset in (c) is the morphological atomic force microscopy for GO distribution. GO are shown as black spots. (e) Ultraviolet photoelectron spectroscopy data of ITO with *h*-GO (red curve) and without *h*-GO (black curve). The work function increased noticeably. (f) X-ray photoelectron spectroscopy spectra for the confirmation of the interaction between the ITO surface and GO nanosheets (the green, blue, and red curves are denoted by O3, O2, and O1, respectively). (g) Schematic of atomic bonding between GO and ITO. This bonding induces charge transfer from ITO to GO.

A GaN-based UV-LED was grown by metal-organic chemical vapor deposition. We used a commercially optimized UV-LED structure with an AlGaN carrier blocking layer^11^ and Mg-doped graded p^++^ layers to achieve an improved hole injection efficiency, as illustrated in Fig. 1a. Mg content in *p*-GaN reached as high as ~10^20^ cm^−3^ (see Supplementary Information, Figure S1). Next, the prepared GO nanosheet solution was spin-casted onto the UV-LED^12^. The GO nanosheets possess hydroxyl, phenol, and epoxy groups in the basal plane, and carbonyl and carboxyl groups on the edge sites. These oxygen functional groups possess negative charges on the GO nanosheets, which induce strong dipole field^13^,^14^. The final structure including the GO nanosheets is shown in Fig. 1a. Using the conventional LED fabrication technique, we obtained a full chip with GO/ITO as a transparent conducting layer and Cr/Au as a p–n-type electrode, as shown in Fig. 1b.

The GO nanosheets of an area of approximately 1 μm^2^ and a thickness of 1 nm, were distributed at relatively low density (referred to as *l*-GO) owing to hydrophobic nature of the pristine ITO layer, as shown in Fig. 1c. The inset in Fig. 1c is the atomic force microscopy image of graphene oxide sheets on the ITO surface. In addition, these were revealed as dark regions compared to the bare ITO layer. To increase the density of GO nanosheets and to achieve uniform distribution on the substrate, the ITO surface was treated with O_2_ plasma prior to GO spin-casting, which increases hydroxyl groups on the surface^15^. In Fig. 1d, high density GO nanosheet was obtained with improved uniformity (see Supplementary Infromation, Figure S2), hence referred to as *h*-GO.

We conducted ultraviolet photoelectron spectroscopy to observe the charge distribution near the surface of ITO passivated with *h*-GO. As depicted in Fig. 1e, the work function of ITO increased considerably from 4.3 to 4.55 eV by passivation of the *h*-GO. The value of work function was estimated from the secondary electron cutoff using the relation Φ = hν-(E_F_-E_cutoff_), where hν, E_F_, and E_cutoff_ are the photon energy of the excitation light (21.22 eV), the Fermi level edge, and the measured secondary electron cutoff, respectively^16^. This results in charge transfer from ITO to GO. O1s spectra from X-ray photoelectron spectroscopy revealed three oxygen-related peaks (Fig. 1f). The O1 and O2 peaks in the inset originate from bulk In_2_O_3_ near ~530.0 eV and O-deficient “sub-oxide” sites associated with O vacancies near ~531.3 eV. The O3 peak near 532.4 eV is related to hydroxyl groups^17^. This peak position was unchanged with GO passivation but the intensity was increased by approximately 5 times (see Supplementary Information, Figure S3). This indicates hydroxyl groups strongly adhere to ITO. The consequence of charge transfer and adhesion is schematically drawn in Fig. 1g. Large D-band near 1,350 cm^−1^ and G-band near 1,620 cm^−1^ did not appreciably change after deposition^18^ (see Supplementary Information, Figure S4).

**Figure 2:**
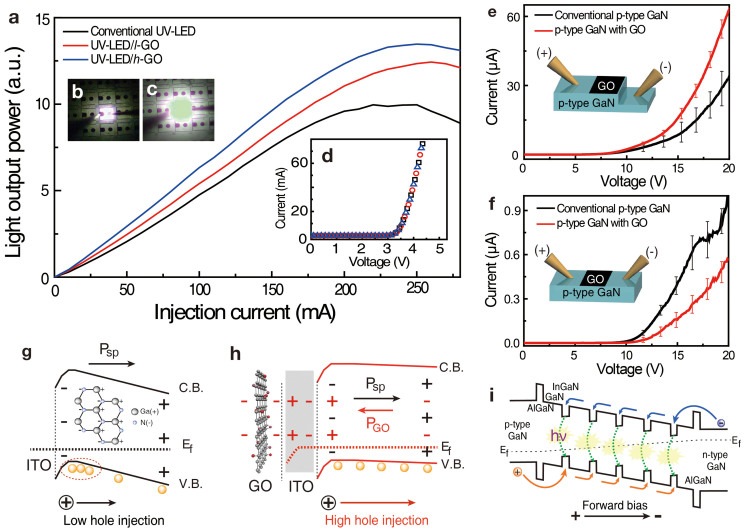
(a) The light output power versus injection current of conventional (black curve), *l*-GO-passivated (red curve), and *h*-GO-passivated (blue curve) UV-LEDs. The light output power increased by approximately 60% when the conventional UV-LED was passivated with *h*-GO. Photograph of UV-LED/*h*-GO of the electroluminescence at an injection current of (b) 1 mA and (c) 5 mA. (d) I-V characteristics of the devices used in (a). No significant change in the samples was observed. I-V characteristics of bare *p*-GaN with and without GO nanosheets measured by (e) vertical and (f) in-plane configuration. Higher (lower) current in vertical (in-plane) configuration was observed from *p*-GaN with GO compared to that without GO. Schematic energy band diagram of *p*-GaN (g) without and (h) with *h*-GO, which explains the suppression of spontaneous polarization. The direction of polarization induced by GO is opposite to the spontaneous polarization. (i) Schematic energy band diagram of active region in UV-LED with applied forward bias.

The light output powers of conventional UV-LED, UV-LED/*l*-GO, and UV-LED/*h*-GO were measured as a function of injection current using a probe station and photodiode detector to investigate the effect of GO nanosheets (Fig. 2a). The light output power of the UV-LED increased linearly at low current and saturated and reduced at high current, called as efficiency droop, due to the insufficiently activated hole concentration or electron over flow, which is a typical light output power behavior^19^,^20^. On the other hand, the light output power of UV-LED/*l*-GO was enhanced by approximately 40% for a typical operating current of 20 mA and almost twice at the saturated current region compared with that of conventional UV-LED without GO nanosheets. This positive contribution was more significant for UV-LED/*h*-GO, which exhibited a 60% increase in light output power at 20 mA. In both cases, the saturated current was prolonged to large values. This can be attributed to the improved hole injection rate in *p*-GaN region by the strong dipole field induced from the GO layers. Images of the electroluminescence emission of UV-LED/*h*-GO at 1 and 5 mA are shown in Fig. 2b and 2c, respectively. The electroluminescence emission is uniform and sufficiently bright.

Enhanced light output power in GaN-based UV-LEDs is generally attributed to improvement in electrical properties, enhancement in light extraction efficiency, increase in the hole injection rate between ITO and *p*-GaN, and an increase in the hole concentrations inside active layer of LED^21^,^22^,^23^. In our samples, the current-voltage (I-V) characteristics and light output power were measured simultaneously, as plotted in Fig. 2d. The I-V curves for the all three samples were nearly identical. Although the work function of ITO increased by 0.25 eV, this only allows to reduce Schottky barrier height for hole injection. Since the I-V of the device is dominated by electron current, this contribution is negligible. This confirms that passivation of the GO nanosheet did not affect the series resistance and current level of the device and that the improvement in light output power is not attributed to the change in electrical properties. To examine the effect of GO nanosheet passivation on the light extraction efficiency of the LEDs, transmission spectra were compared (see Supplementary Information, Figure S5). The transmittance of the ITO/*h*-GO was quite similar to that of the ITO without GO nanosheets. This observation confirms that the improvement in the light output power of GO-passivated UV-LEDs is not due to an enhancement in light extraction efficiency.

To investigate the origin of enhanced light output in LED with GO, source-drain hole currents in bare *p*-GaN were measured for both perpendicular and parallel directions to *p*-GaN c-axis. For the measurement, we prepared four pieces of *p*-GaN which is grown by same conditions with *p*-GaN of UV-LED. Before coating GO, two pieces of p-GaN was etched by inductively coupled plasma to measure vertical I-V curves. GO was coated on the center of both samples with same area. Distance of cathode and anode tips were kept for comparing all I-V curves. The vertical source-drain hole current was enhanced with GO passivation (Fig. 2e). This is a direct evidence of the reduced spontaneous polarization, in other words, the negative charges at the surface of *p*-GaN are compensated by the induced positive charges (Figs. 2g and 2h, and supplementary Information Fig. S6). The effect of such charge compensation was also visible in the in-plane source-drain current (Fig. 2f). In this case, due to the gating effect of the reduced negative charges at the *p*-GaN surface, the in-plane hole current was reduced. As a consequence, the hole injection rate is enhanced to supply more hole carriers to the active quantum well region in the real device, giving rise to enhanced light output power in Fig. 2a. Since this is the minority hole current contribution (hole mobility is about 100 times lower than electron mobility in the device), I-V is still dominated by electron current and not modified appreciably, as shown in Fig. 2d^24^. It is also intriguing to see the reduced current fluctuation with GO passivation in both cases. This is ascribed to the enhanced field uniformity in *p*-GaN region due to the random location of GO flakes.

**Figure 3:**
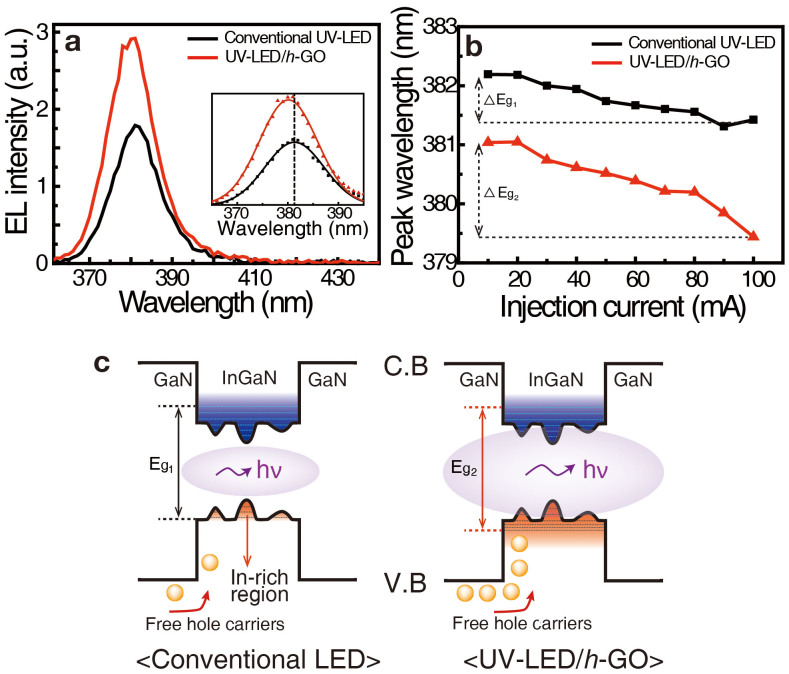
(a) Macro-EL spectra of the UV-LEDs with (red curve) and without (black curve) GO nanosheets measured at 20 mA. The inset displays the Gaussian fitted EL spectra, including the original EL spectra represented by dots. The peak position of the EL spectrum with GO nanosheets is blue-shifted by over 1 nm compared with the conventional UV-LED. (b) Peak positions of the EL spectra with (red line) and without (black line) GO nanosheets as a function of injection current from 10 to 100 mA. The blue shift between 10 and 100 mA with GO sheets is more pronounced than that with the conventional one. (c) Schematic of the band diagram of active InGaN/GaN MQW layers with (right side) and without (left side) GO nanosheets.

The Ga-faced c-plane GaN has, in general, spontaneous polarization from top to bottom inherently due to dipole-like N-Ga arrays that is typical for GaN crystal grown on *c*-plane sapphire substrate^4^. Local charge distributions in each layer near Ga-faced *p*-GaN are depicted in the inset of Fig. 2g. In this arrangement, the hole carriers in the valence band of *p*-GaN are bound by potential valleys at the ITO/*p*-GaN interface that are induced by a tilted energy band, thus suppressing hole injection rate to the active layer. In this study, GO passivation creates dipole field outside ITO/*p*-GaN layer and induces positive charges (indicated by red color) at the top surface of *p*-GaN region. This dipole field (P_GO_) is opposite of the spontaneous electric polarization (Fig. 2h). Because of the suppressed internal polarization field in *p*-GaN region with GO passivation, the hole injection rate to active layer is improved. Hole carriers are therefore injected more efficiently into active layer so as to increase electron-hole radiative recombination, contributing enhanced light output power, as observed in Fig. 2a. This concept is schematically demonstrated in Fig. 2i. To examine enhancement of the hole concentration in the active layer, electroluminescence spectrum was obtained at room temperature by collecting all the lights with lens from the whole device (Fig. 3a). The intensity of UV-LED/*h*-GO was increased by about 60% compared to that without GO by the increased hole carrier concentration in the active layer as a consequence of enhanced hole injection rate in the *p*-GaN transport layer. The peak position shift in the EL spectra of the conventional UV-LED was about 0.8 nm (68 meV) at 100 mA, known as the conduction band filling effect^25^. This should be distinguished from die-to-die variation in the same wafer, since the EL was measured by varying injection current in the same device, although the fluctuation is in the same order of magnitude. This shift in UV-LED/*h*-GO was amplified to 1.6 nm (137 meV) at 100 mA^26^. This additional blueshift is ascribed to the valance band filling effect by the enhanced hole carrier concentration in the MQWs^27^. The enhanced hole carrier injection rate in *p*-GaN supplies more hole carriers to lead to valence band filling in the active layer (Fig. 3c). Because the number of hole carriers injected into the MQWs is small in the conventional UV-LED, radiative recombination primarily occurs in In-rich region, where the band gap (E_g1_) is minimum in the QW^28^. On the other hand, in the UV-LED/*h*-GO, more hole carriers can contribute to radiative recombination throughout all the regions of the device, including both In-rich and In-poor regions (E_g2_), resulting in a blue-shift in the EL spectrum and emission uniformity improvement.

**Figure 4:**
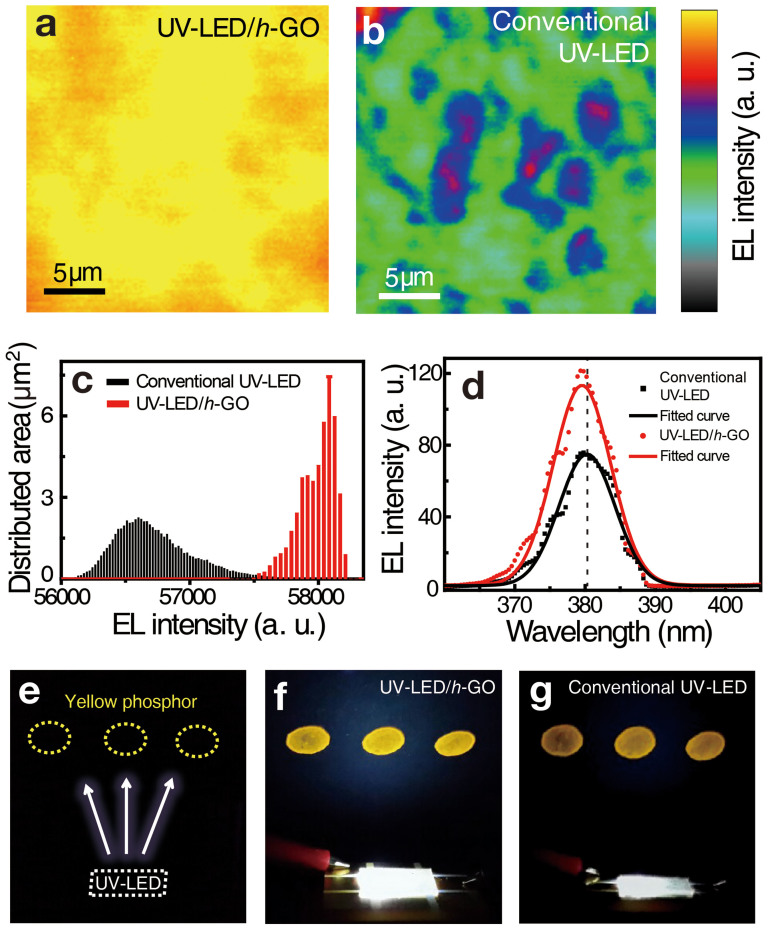
CSEM images of GaN-based UV-LEDs (a) with and (b) without *h*-GO measured at a current of 5 mA. The EL intensities with *h*-GO are more uniform and higher than those of the conventional one. (c) Histogram of the EL intensities for the scanned areas of the UV-LEDs with *h*-GO (red line) and without *h*-GO (black line). (d) The local EL spectra at specific positions are indicated by dotted circles in (a) and (b). The blue shift of the EL peak is attributed to the band-filling effect in localized states inside InGaN/GaN MQWs. (e) Optical image of fully fabricated UV-LED devices with yellow phosphor pads. Yellow dotted circles indicate yellow phosphor pads and white dashed square is UV-LED. Higher brightness of GO-passivated device at 20 mA in (f) compared to that of the conventional device in (g).

To confirm emission uniformity of the device, we performed two-dimensional confocal scanning electroluminescence microscopy^29^. An injection current of 5 mA was fixed during emission measurements for the UV-LEDs with and without *h*-GO nanosheets (Figs. 4a and 4b). For the UV-LED with *h*-GO, not only the emission uniformity was improved but also absolute emission intensity was augmented compared to the UV-LED without *h*-GO, which is confirmed by the broad width in the low intensity region in the conventional UV-LED and narrow width in the high intensity region in the UV-LED/*h*-GO (Fig. 4c). The average EL spectra collected over the entire scanning area presented in Figs. 4a and 4b are shown in Fig. 4d, again revealing the increased areal intensity in the UV-LED/*h*-GO. Overall EL spectra still revealed clearly the blue shift. The similar conduction band-filling phenomenon beyond the localized states inside the InGaN/GaN MQWs by increasing the injection current have been discussed previously^30^,^31^. This improvement in the intensity and uniformity becomes more severe by valence band filling effect at higher injection current limit as shown in Fig. 3b.

Towards the application of white LED, we carried out a fluorescence test of the GO-passivated UV-LED with yellow phosphor. Figure 4e is an optical image of fully fabricated UV-LED with yellow phosphor pads without applied current in UV-LED. In case of GO-passivated UV-LED at 20 mA, Yellow phosphor pads emitted bright fluorescence at 20 mA due to UV light irradiation (Fig. 4f). This fluorescence is significantly brighter than that of the conventional UV-LED at the same current (Fig. 4g). This demonstrates the white LED performance with improved light output power in the GO-passivated UV-LED, which can be further applied for commercialized white LEDs as well as solar cells, and laser diode. In such applications, a degradation of GO by UV irradiation might be a problem. Reminding that such degradation take place only when the GO sheets are exposed in air or chemicals, protecting GO by conventional encapsulation should be enough, which is discussed in detail in Supplementary information (see Supplementary Information, Figure S7).

In addition, THz transmission were measured to determine change of hole concentration in the *p*-GaN (see Supplementary Information, Figure S8). The transmission amplitude of ITO/*p*-GaN/GO decreased by 6.5% in average compared to ITO/*p*-GaN. This increased absorption means conductivity increase, which implies that the carrier density is increased. From these results, we observed that improved hole injection rate induces an increase of free carrier concentration in the *p*-GaN.

## Conclusion

We have shown that the spontaneous polarization was suppressed by simply passivating GO on top of the fully fabricated UV-LED chips under ambient conditions. As a consequence, the hole injection rate in p-GaN was increased. It leads to augmented electron-hole radiative recombination in active layer so as to enhance the light output power of GaN-LED device up to 60%. No further treatment is required. GO solution is easily obtained with cheap cost. We believe that our simple method of passivation with GO is a breakthrough for further augmenting the emission efficiency in UV-LEDs.

## Methods

### UV-LED fabrication

Trimethylgallium, trimethylindium, and ammonia were employed as precursors for Ga, In, and N, respectively. H2 was used as the carrier gas, and N2 gas was used as the carrier gas for the InGaN, MQWs, and GaN barrier layers. A 20 nm thick GaN nucleation layer, a 2 µm thick undoped GaN layer, a 2-µm-thick n-type GaN layer, 5 pairs of InGaN/GaN MQW active layers, p-type AlGaN carrier blocking layer, a 150 nm thick p-GaN layer, and graded p++GaN layers were subsequently grown on a c-plane sapphire substrate (see Supplementary Information, Figure S1). Following epitaxial growth, hole carriers of p-GaN were activated by thermal annealing process at 940°C for 40 s. Then, the surface of the LED epitaxial layers was partially etched down to the n-type GaN layer using inductively coupled plasma etching with Cl2/BCl3/Ar plasma. A 200 nm thick ITO layer was deposited as a transparent conductive layer. Using a conventional LED fabrication technique, we produced a full chip with ITO as the transparent top conductive layer and Cr/Au as the p–n-type electrode, as shown in the top of Fig. 1b.

### Preparation and passivation of the the GO nanosheets to UV-LED

GO nanosheets were prepared by using a modified Hummers method. Natural graphite was purchased from Alfa Aesar, 99.999% purity, 200 mesh. Natural graphite of 5 g was dissolved in 350 ml of 10 M H2SO4. KMnO4 of 15 g was slowly added in the solution over approximately an hour. Stirring was continued for two hours in an ice-water bath. To obtain highly oxidized graphite, the mixture was stirred vigorously for three days at room temperature. De-ionized water was subsequently added, followed by the stirring for 10 min. Aqueous solution of H2O2 (30 wt%) was then added, and the mixture was stirred for 2 h at room temperature. Aqueous solution of HCl (35 wt%) was then added and stirred for 30 min at room temperature. After the supernatant solution was decanted, deionized water was slowly added and stirred for 30 min. The graphite oxide solution of 1 g/l in water was sonicated for an hour to exfoliate the GO nanosheets. To obtain highly dispersed GO, centrifugation at 10,000 rpm was performed for an hour, and the supernatant solution was decanted. The prepared GO solution was spin-casted onto the ITO surface at 4,000 rpm for 10 s. To achieve a high density and uniform distribution of GO nanosheets on the ITO surface, the ITO surface was treated with O2 plasma for 5 min. The hydrophilicity of ITO was enhanced, as indicated by the contact angle measurement (Surface Electro Optics/Phoenix 300, Fig. S2). As a result, the *h*-GO nanosheet was uniformly distributed on the ITO surface without a concomitant loss of transmittance relative to the pristine ITO and *l*-GO/ITO, as indicated in Fig. 2e.

### Characterization of the GO/GaN-based LED

Raman scattering measurements were performed using a micro-Raman system with He-Ne laser (633 nm). I-V curves were measured using a conventional probe station system equipped with a Keithley source meter 2400. Transmittance spectra were measured on a JASCO model V-560 at room temperature. ultraviolet photoelectron spectroscopy data (Kratos model AXIS-NOVA) were obtained with a helium discharge lamp at 7.9 × 10^−9^ Torr. To confirm the change in the carbon to oxygen atomic ratio in the *h*-GO, X-ray photoelectron spectroscopy analysis was performed using a Multilab2000 (Thermo VG Scientific Inc.) spectrometer with monochromated Al Kα X-ray radiation as the X-ray excitation source. To generate THz pulses, a home-made fs laser with an approximately 30 fs pulse width and 80 MHz repetition rate with a center wavelength of 800 nm was irradiated into a commercially available photoconductive antenna (Batop GMBH). The generated THz pulse ranged from 0.1 to 2.5 THz. The THz pulses transmitted through the sample were detected by the electro-optic sampling method. CSEM images were obtained by modified optical confocal microscopy equipped with a PZT nano-positioner, an optical fiber, a power supplier, and synchronized external signals. Details have been provided in previous report29. In addition, morphologies and thicknesses of the GO nanosheets were imaged by field emission scanning electron microscopy (S-4700, Hitachi) and atomic force microscopy (NTEGRA SPECTRA, NT-MDT).

## Author Contributions

H.J., S.Y.J., and D.J.P. contributed to this work in experiment planning, experiment measurements, data analysis and manuscript preparation. The correlation between theoretical and experimental results was discussed with J.T.H. and H.J.J. The fabrication of the LED device was carried out by H.J.J. and E.-K.S. The THz transmission measurements were performed by S.J.P. and Y.H.A. Graphene oxide nanosheets were synthesized by S.J. Scanning electron microscopy and atomic force microscopy were performed by S.Y. and H.Y.K. The measurement and analysis of X-ray and ultraviolet photoelectron spectroscopies were carried out by K.J.B. and G.-W.L., Y.H.L. and M.S.J. contributed to experiment planning, data analysis and manuscript preparation.

## Supplementary Material

Supplementary Information Supplementary Information
