# Supplementary material for: Suppressing spontaneous polarization of p-GaN by graphene oxide passivation: Augmented light output of GaN UV-LED
Source: Sci Rep. 2015 Jan 14;5:7778. doi: 10.1038/srep07778 (PMC4293595; doi:10.1038/srep07778)
Supplement: Supplementary Information [file srep07778-s1.doc]

Supplementary Information

Suppressing spontaneous polarization of p-type GaN by graphene oxide passivation: Augmented light output of GaN UV-LED

Hyun Jeong^1,5,†^, Seung Yol Jeong^2,†^, Doo Jae Park^1,†^, Hyeon Jun Jeong^1,6^, Sooyeon Jeong^2^, Joong Tark Han^2^, Hee Jin Jeong^2^, Sunhye Yang^2^, Ho Young Kim^2,5^, Kang-Jun Baeg^2^, Sae June Park^3^, Yeong Hwan Ahn^3^, Eun-Kyung Suh^4^, Geon-Woong Lee^2,*^, Young Hee Lee^1,6,*^, and Mun Seok Jeong^1,6,*^

*Correspondence to [mjeong@skku.edu, gwleephd@keri.re.kr, leeyoung@skku.edu]

^1^Center for Integrated Nanostructure Physics (CINAP), Institute for Basic Science (IBS), Sungkyunkwan University, Suwon 440-746, Republic of Korea, ^2^Graphene Hybrid World Class Laboratory, Nano Carbon Materials Research Group, Korea Electrotechnology Research Institute, Changwon 641-120, Republic of Korea, ^3^Department of Physics and Division of Energy Systems Research, Ajou University, Suwon 443-749, Republic of Korea, ^4^School of Semiconductor and Chemical Engineering, Semiconductor Physics Research Center, Chonbuk National University, Jeonju 561-756, Republic of Korea, ^5^Laboratoire de Nanotechnologie et d’Instrumentation Optique, Institut Charles Delaunay, CNRS-UMR 6279, Université de Technologie de Troyes, BP 2060, 10010 Troyes, France, ^6^Department of Energy Science, Sungkyunkwan University, Suwon 440-746, Republic of Korea

**S1. Secondary ion mass spectroscopy for UV-LED characterization**

To guarantee the quality of the p-type GaN used in this study, the incorporation of Mg dopant was verified by secondary ion mass spectroscopy (SIMS). The measurements were performed with an impact energy of 7.5 keV, a current of 50 nm, a raster size of 200 × 200 μm, and an analysis diameter of 60 μm. Figure S1 presents the SIMS data for Mg (black line), Al (red line), and In (blue line) in the LED structure. The depth profile of SIMS from 100 to 200 nm revealed that the average Mg concentration was approximately 3 × 10^20^ atomic/cm^-3^, which is comparable to that found in industrially manufactured LEDs. The Mg concentration near the ITO/p-type GaN interface decreased gradually with increasing depth, indicating that graded doping was achieved to improve the hole injection rate of the InGaN/GaN multiple quantum wells (MQWs). In addition, the Al concentration was considerably high for p-type GaN/MQWs and n-type GaN/MQWs, confirming the presence of AlGaN carrier blocking layers for preventing carrier overflow.


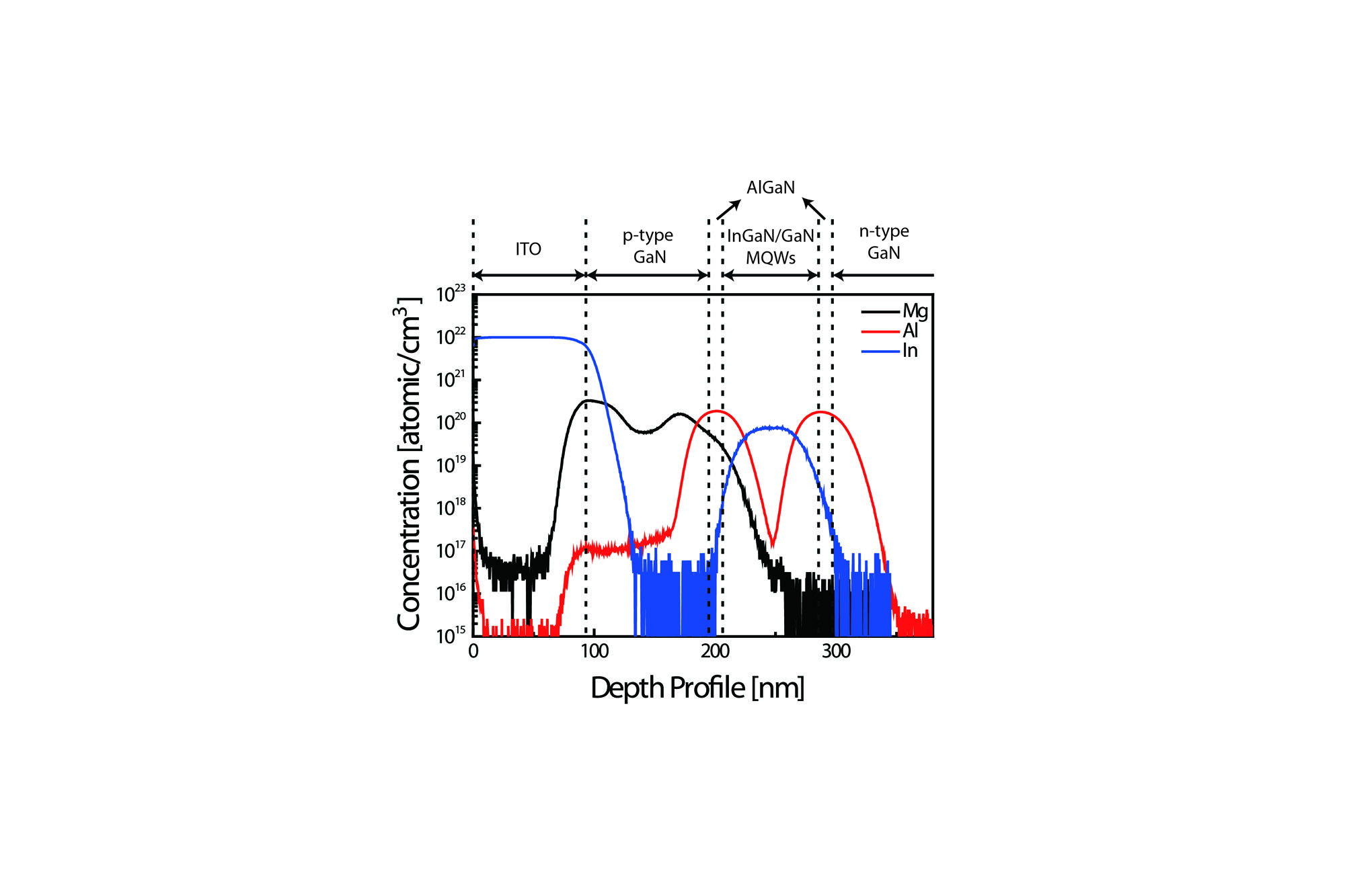


Figure S1. Depth profiles of the Mg, Al, and In concentrations measured by secondary ion mass spectroscopy (SIMS) for the UV-LED structure used in this study.

**S2. Water contact angle measurements for examining the hydrophilicity of the O_2_ plasma treated h-GO/ITO surface**

To characterize the hydrophilicity of the surfaces with respect to the bare ITO, ITO/GO, and O2 plasma-treated ITO/GO, the water contact angle was measured as shown in Figure S2. The contact angles were 86.46°, 52.23°, and 10.39°, respectively. Increasing contact angles indicate higher surface wettability, as a result of hydrophilic properties. Comparing these samples, the O2 plasma-treated ITO/GO exhibited a higher contact angle than the other surfaces, confirming its higher hydrophilicity. In addition, the GO nanosheets were uniformly coated on the substrate following hydrophilic treatment of the surface.


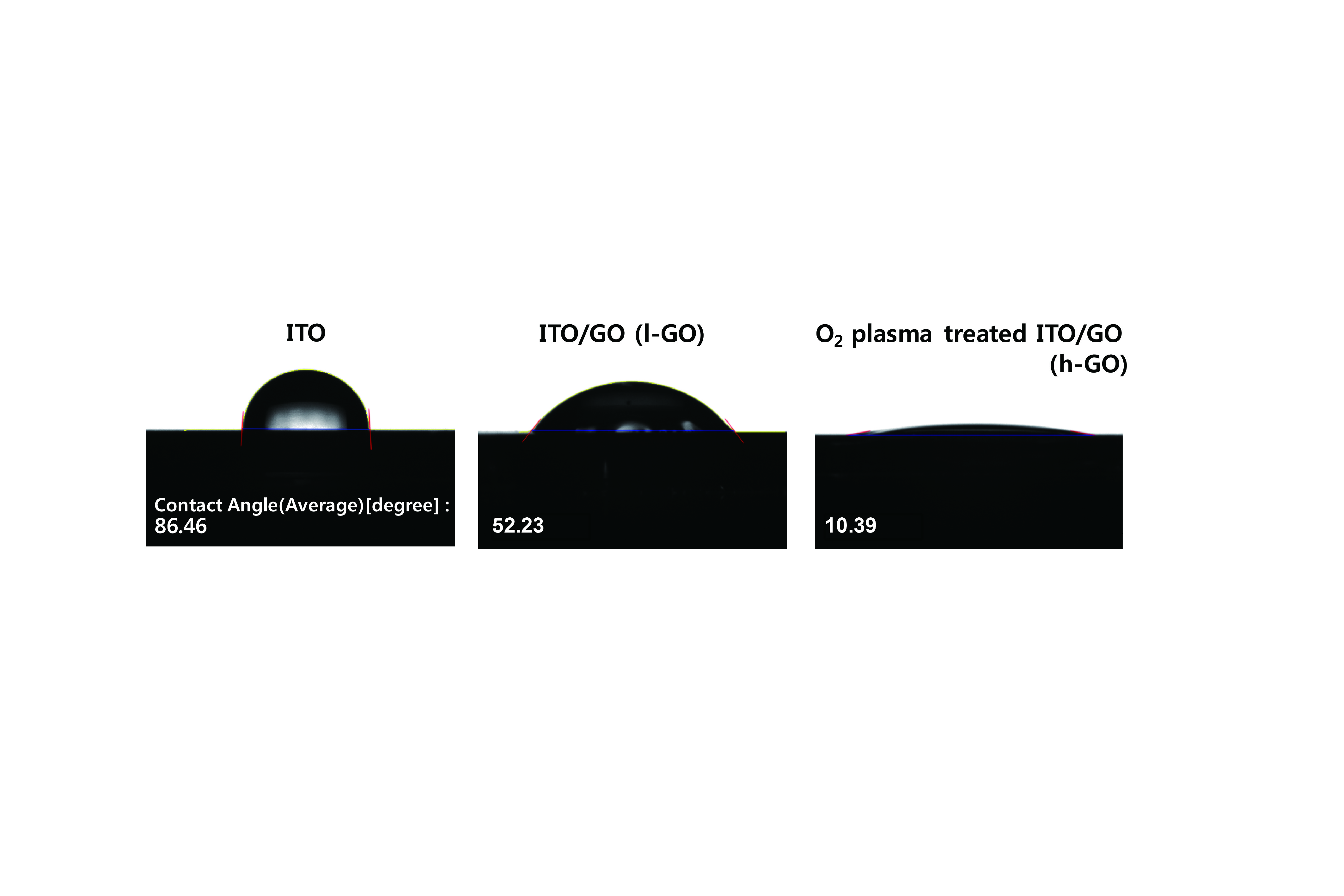


Figure S2. Static water contact angles of bare ITO, ITO with l-GO, and ITO with h-GO. The density of the GO nanosheets was determined by the hydrophilicity of the substrate.

**S3. XPS and FTIR analysis to confirm atomic bonding of the GO nanosheet to ITO**

For the quantitative analysis of each functional group in the GO, a multiple Gaussian and Lorentzian fit was applied to the x-ray photoelectron spectroscopy (XPS) spectrum as a function of binding energy (black curve). The proportion of oxygen functional groups on the GO nanosheets (C-O peak, blue curve, hydroxyl and epoxy groups, approximately 287.6 eV) was higher than that of the C-C=C groups (red curve, *sp*2 and *sp*3, approximately 284.6 eV) in the C1s peak, indicating a high concentration of hydroxyl and epoxy groups. Moreover, various oxygen functional groups were described, including C=O (green curve, carbonyl, approximately 288.3 eV) and O-C=O (yellow curve, carboxyl, approximately 289.1 eV). In addition, Fourier Transform Infrared (FTIR) spectroscopy confirmed the atomic bonding of the GO nanosheets to the basal plane and edge sites. The FTIR spectra revealed the presence of various oxygen functional groups on the GO nanosheets, including alcohols, phenols, carboxyls, and epoxides. Correspondingly, as described by the atomic ratio of the O1s peak in the XPS spectra, the O3/O1 peaks indicate saturated coverage of OH groups on the ITO surface. The ratio of amount of GO to ITO (1.4) was approximately 5 times higher than that of the bare ITO (0.3). These results confirm the significant increase in OH groups on the substrate owing to the oxygen functional groups on the basal plane of the GO nanosheets.

**
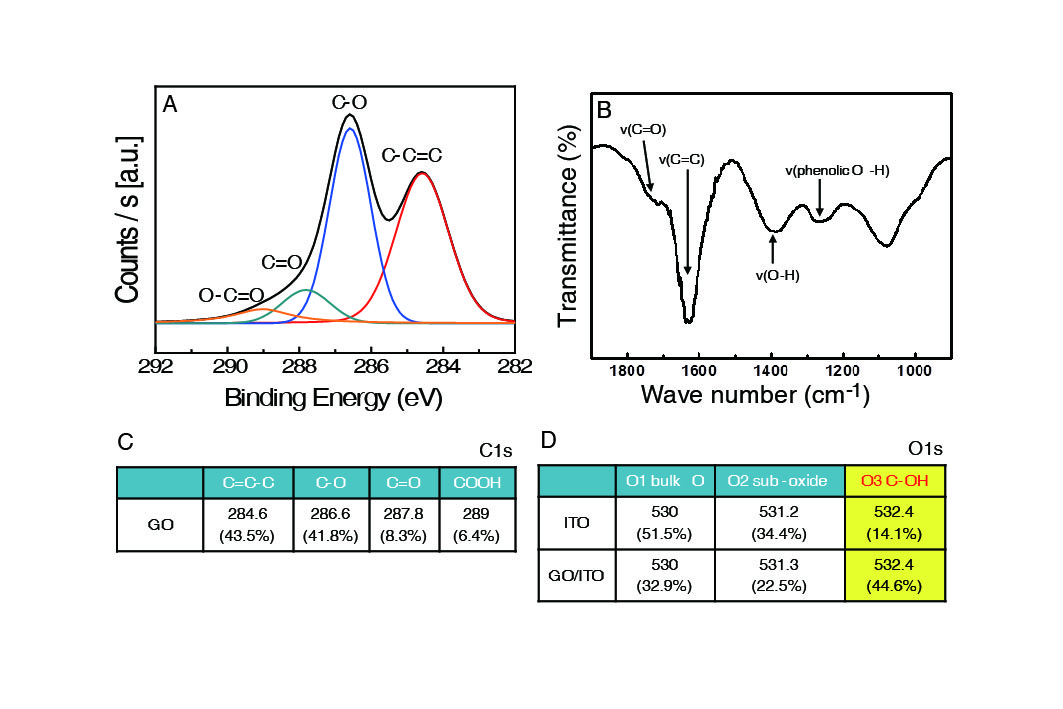
**

Figure S3. (a) XPS and (b) FTIR spectrum of GO nanosheet. The amount and proportion of (c) C1s and (d) O1s of GO nanosheet extracted from XPS spectrum.

**S4. Micro-Raman spectroscopy of *h*-GO/ITO**

The Raman spectra shown in Figure 1g also indicate the presence of GO on ITO. The D-band near 1,350 cm^-1^ is prominent in the *h*-GO-coated ITO film (red curve in Figure 1g) owing to the presence of oxygen functional groups and structural defects on the GO nanosheets.


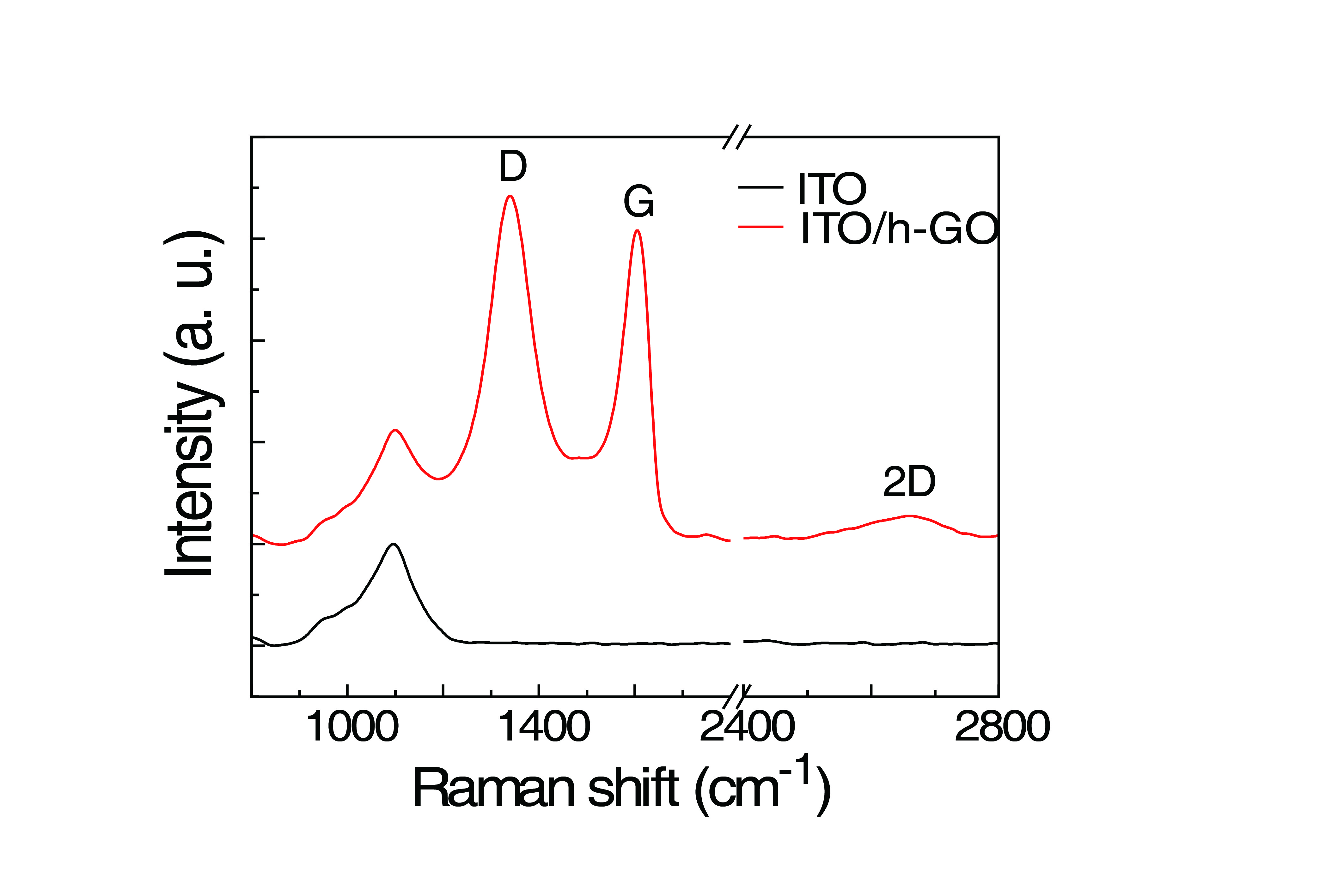


Figure S4. Raman spectra of ITO with and without GO nanosheets are plotted in red and black, respectively.

**S5. UV/VIS transmittance of ITO with and without GO**

To examine the effect of GO nanosheet passivation on the light extraction efficiency of the LEDs, transmission spectra were compared. The transmittance of the ITO/h-GO was similar to that of the ITO without GO nanosheets. At a device operating wavelength of 380 nm, the transmittance decreased only by approximately 0.2%. This observation confirms that the improvement in the light output power of GO-passivated UV-LEDs is not due to an enhancement in light extraction efficiency.


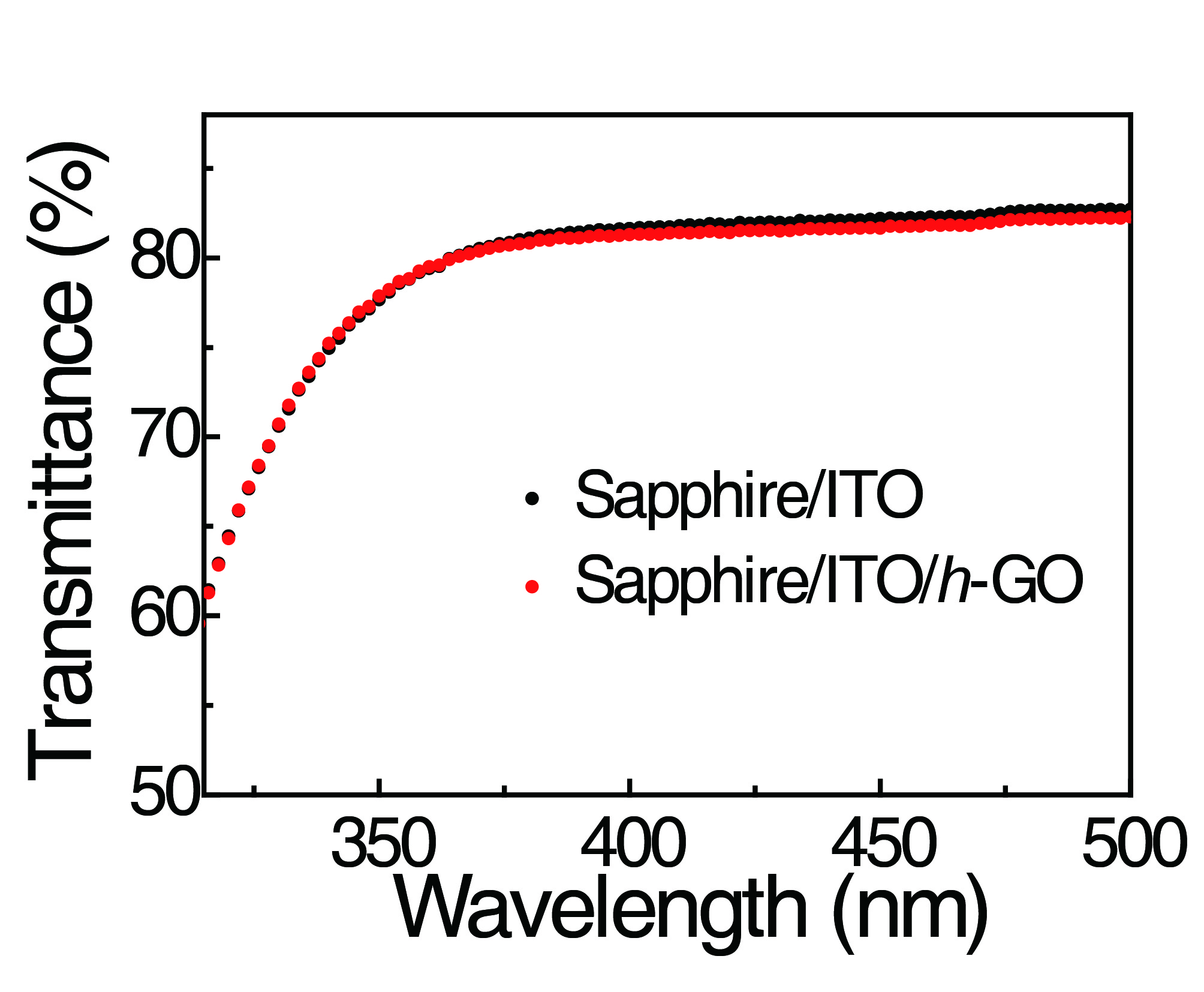


Figure S5. Transmittance spectra of sapphire/ITO with h-GO (red curve) and without h-GO (black curve). The change in the transmittance between two samples was negligible.

**S6. Current-voltage characteristics of p-type GaN**

For supporting the polarization induced by GO in p-GaN surface, we measured source-drain current of p-type GaN in parallel directions to p-type GaN c-aixs, as shown in Figure 2f. The in-plane source-drain current of p-GaN with GO was smaller than that without GO. The reduced current of p-type GaN with GO can be explained by the restrained gate effect on the surface, which occurred by spontaneous polarization, as depicted in Figure S6. Finally, due to accumulated positive charges at the surface of p-type GaN, which compensate built-in negative charges, the in-plane hole current was reduced in the presence of GO compared to that without GO.

**
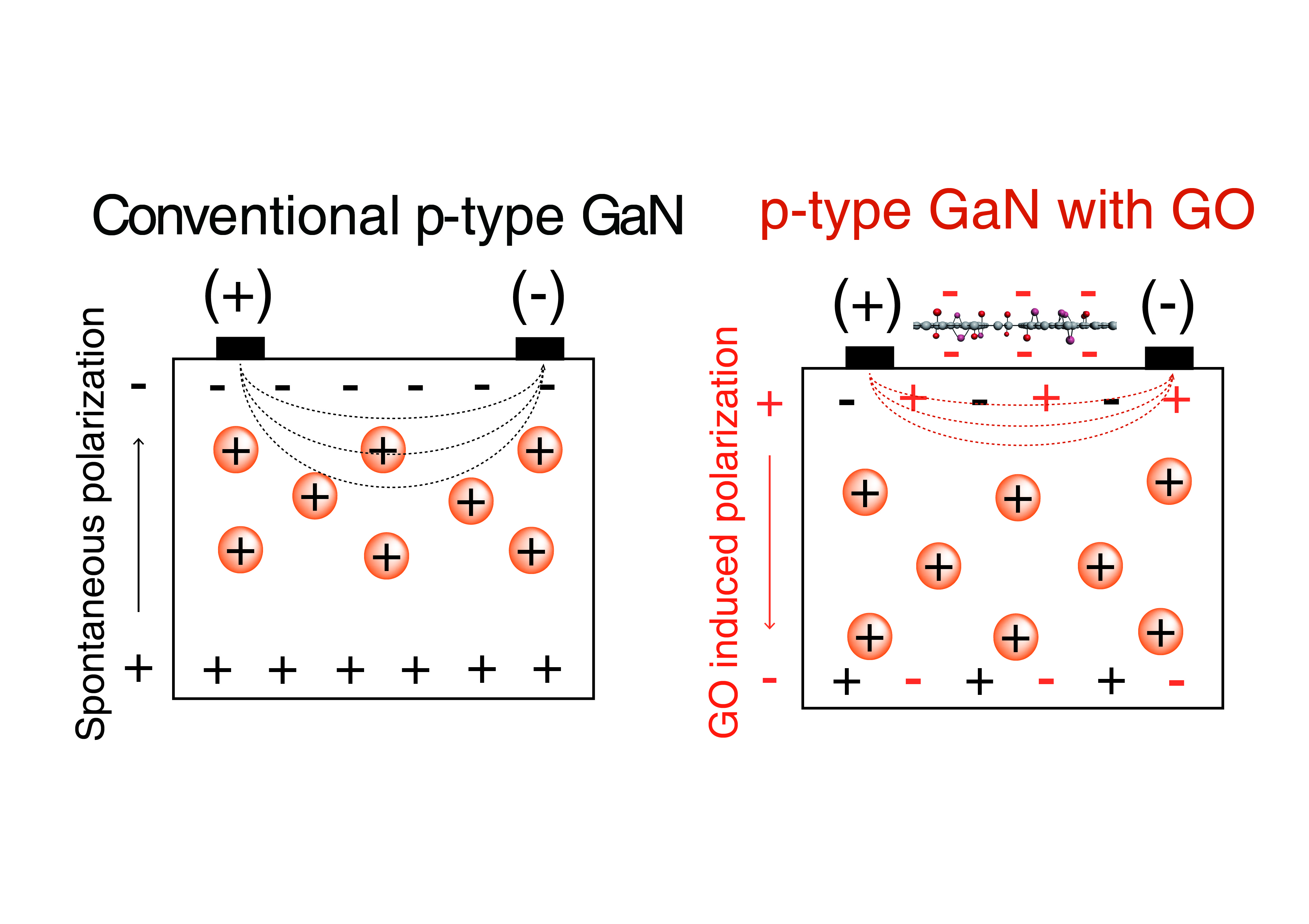
**

Figure S6. Schematic of hole current injection by lateral configuration. In-plane current flow of p-type GaN with GO is restricted by GO-induced electric polarization.

**S7. Absorption spectra for verification of stability of UV-LED with GO**

To verify this, we have performed a UV light irradiation with long time scale of 24 hours and have observed whether the performance of LED would change or not. Two kinds of samples are prepared in this experiment: Bare graphene oxide (GO) film on sapphire substrate and GO film with silicone resin encapsulation (Dow corning) on sapphire substrate. We used a 355 nm (3.6 eV) solid state laser as an UV source which has higher energy than emission energy of our UV-LED (380 nm, 3.25 eV). Samples were irradiated for 24 hours in ambient condition, while power of irradiation source was kept 3 mW which is comparable to UV-LED at driving current of 20 mA. Fig. S7 (A) is absorption spectrum of bare GO with and without irradiation treatment. In bare GO with irradiation, absorbance value at 250 nm which indicates amount of reduction is about 50% increased, compared to sample without irradiation. However, in encapsulated GO with irradiation, absorbance at 250 nm is only 0.8% increased, compared to sample without irradiation as shown in Fig. S7 (B). Since the value of 0.8% is within error range for absorption measurement, we can conclude that encapsulated GO is not influenced by UV light irradiation. Different shape of absorption spectra between exposed GO and encapsulated GO is attributed by absorption of silicone encapsulants.


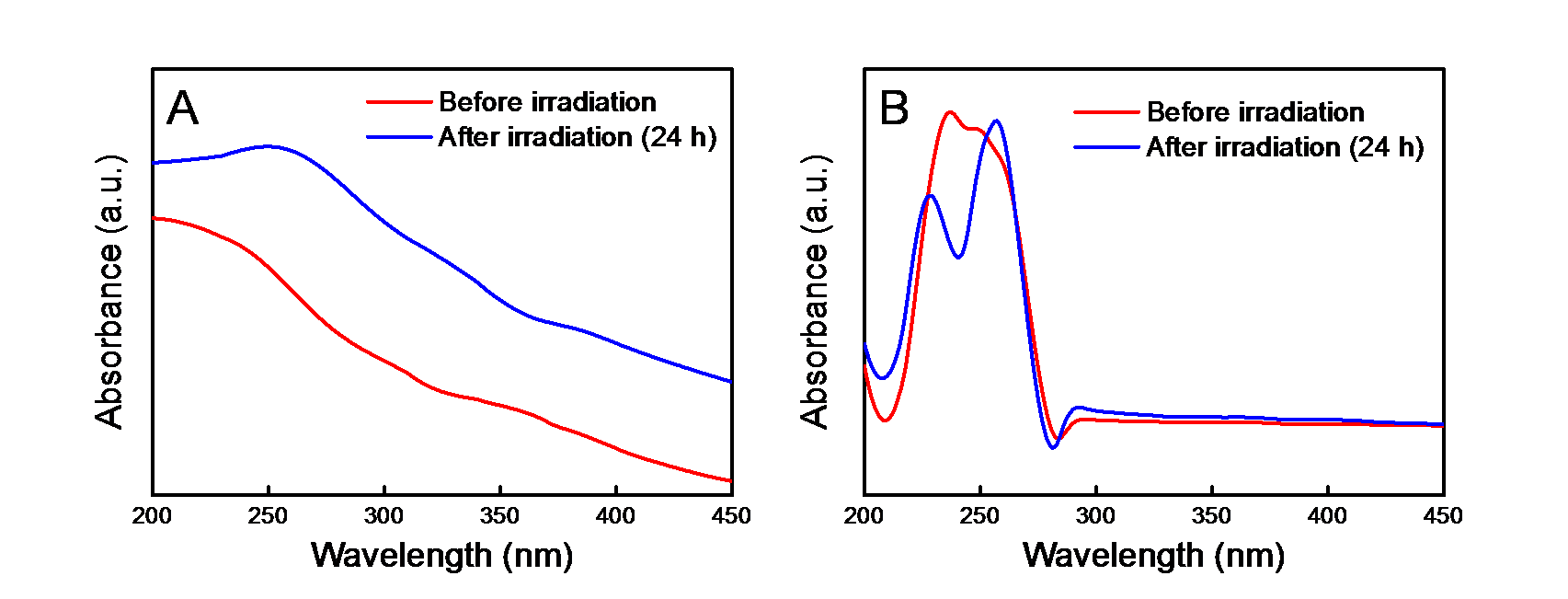


**Figure S7**. Absorption spectra of (A) bare GO and (B) encapsulated GO on sapphire. Almost same absorbance value at 250 nm is observed in GO encapsulated by Si resin with and without UV irradiation.

**S8. THz transmittance and increase of free hole concentration**

To test the effect of the hole concentration in p-type GaN region, the transmittance in the THz frequency range was observed using a conventional THz time-domain spectroscopy technique. The reference sample was grown to AlGaN layer from sapphire substrate to see exclusively for this test and thus the difference includes only the effect of ITO/p-type GaN. Since ITO carrier concentration does not rely on the polarization, the change in the THz dynamics is negligible and therefore the change in dynamics is solely ascribed to the change in p-type GaN. The transmission amplitude decreased by 6.5% in average over the observed THz range (0.5-2.0 THz) in ITO/p-type GaN/*h*-GO (Figure S7a), in good contrast with the negligible change in optical transmission in **Figure 2**e. This increased absorption is equivalent to conductivity increase. This strongly implies that the carrier density in p-type GaN hole-injection layer increased. This argument is supported well by the dielectric function extracted from the complex THz transmission spectra, and by the considerable change of the plasma frequencies obtained by Drude fits on the dielectric function of p-type GaN with h-GO and without h-GO.

The dielectric function of the thin film can be extracted from the amplitude and phase information of the complex THz transmission. Figure S7b presents the real part of the dielectric constant of p-type GaN with *h*-GO nanosheets As observed in this figure, the real part of the dielectric constant follows Drude-like behavior, which denotes that the induced polarization was primarily governed by free holes. A fit based on the Drude model was performed to obtain the plasma frequency *ω_p_* for the p-type GaN with and without *h*-GO, revealing frequencies of 44.4 and 53.8 THz, respectively. Because the plasma frequency is proportional to the square root of the carrier concentration *n*, i.e., $\omega_{p}\propto n$, this considerable difference confirms that the hole concentration was effectively increased by the *h*-GO coating by approximately 47%.


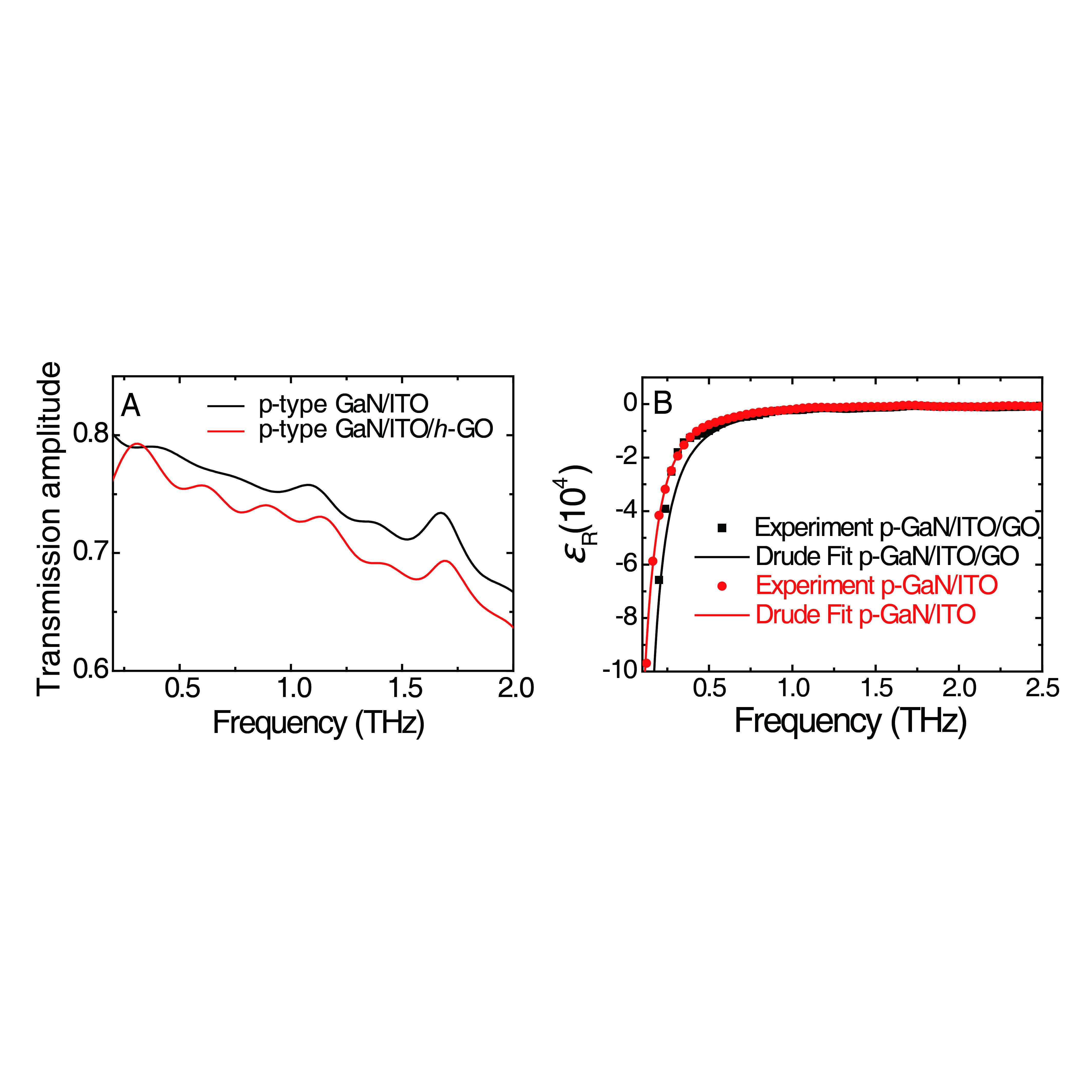


**Figure S8**. (A) THz transmittance spectra of the p-type GaN/ITO with GO nanosheets (red curve) and without GO nanosheets (black curve). (B) The real part of the dielectric function of p-type GaN coated with *h*-GO extracted from the complex THz transmission amplitude.
